# Bilateral anomalous drainage of the posterior divisions of renal veins into the azygos venous system in a 20-year-old woman: a case report

**DOI:** 10.1186/s13256-016-1134-x

**Published:** 2016-12-03

**Authors:** Pedro Pallangyo, Frederick Lyimo, Paulina Nicholaus, Stephano Masatu, Mohamed Janabi

**Affiliations:** 1 Department of Adult Cardiovascular Medicine, The Jakaya Kikwete Cardiac Institute, P.O Box 65141, Dar es Salaam, Tanzania; 2 Department of Radiology, Muhimbili National Hospital, P.O Box 65000, Dar es Salaam, Tanzania

**Keywords:** Renal vein anomaly, Renal vein variations, Azygos venous system, Azygos vein, Hemiazygos vein, Tanzania

## Abstract

**Background:**

Renal vein anomalies are relatively infrequent and generally asymptomatic. Preoperative knowledge of such variants is, however, of paramount importance in several angiographic and surgical procedures including renal venography, renal vein sampling, spermatic embolization, and renal transplantation. Inadequate knowledge and failure to recognize such anatomic variations may lead to several operative hazards including hemorrhage, nephrectomy, and even death.

**Case presentation:**

We report a case of bilateral anomalous drainage of the posterior divisions of renal veins into the azygos venous system in a 20-year-old woman of African descent from Tanzania who presented to us with a 12-year history of recurrent anemia. She had anemia, a positive sickling test, and hemoglobin electrophoresis revealed a sickle cell trait (AS). She underwent computed tomography angiography of her chest and abdomen to rule out the presence of arteriovenous malformations. Aortography findings were normal but venography results revealed features of tortuously dilated azygos and hemiazygos veins each receiving blood from its respective posterior division of renal vein.

**Conclusions:**

Although venous anomalies are relatively infrequent and generally lack a clinical significance, a thorough understanding of embryologic development and its associated errors is of immense importance in equipping angiographers and surgeons to select appropriate interventional/operative techniques, anticipate risks, and prevent intervention-related complications.

## Background

Venous embryology is among the neglected basic sciences; however, its potential to explain various ambiguities attributed to anatomic variations cannot be overstated [1]. In the general population, the overall incidence of congenital vascular malformations is approximately 1.5% with a venous predominance of 2:1 [2]. In general, venous anomalies result from errors of embryological development in the venous fetal circulation and are exclusively present from birth. Anomalies of renal veins are relatively rare, usually detected during routine examinations performed for other indications, and often make image interpretation a challenging undertaking [3–5].

Several renal vein anomalies including circumaortic, retroaortic, and rarely supernumerary renal veins have been documented in the literature [6]. Although a majority of these anatomic variations are asymptomatic and lack a clinical significance, inadequate knowledge and failure to recognize them can result in operative hazards including hemorrhage, nephrectomy, and even death [3, 4, 6]. Despite numerous previous publications regarding the variants of renal veins, we did not find any that resemble the anomaly reported here, even after an extensive literature search. We report a case of bilateral anomalous drainage of the posterior divisions of renal veins into the azygos venous system in a 20-year-old woman from Tanzania who presented with a 12-year history of recurrent anemia.

## Case presentation

**Fig. 1:**
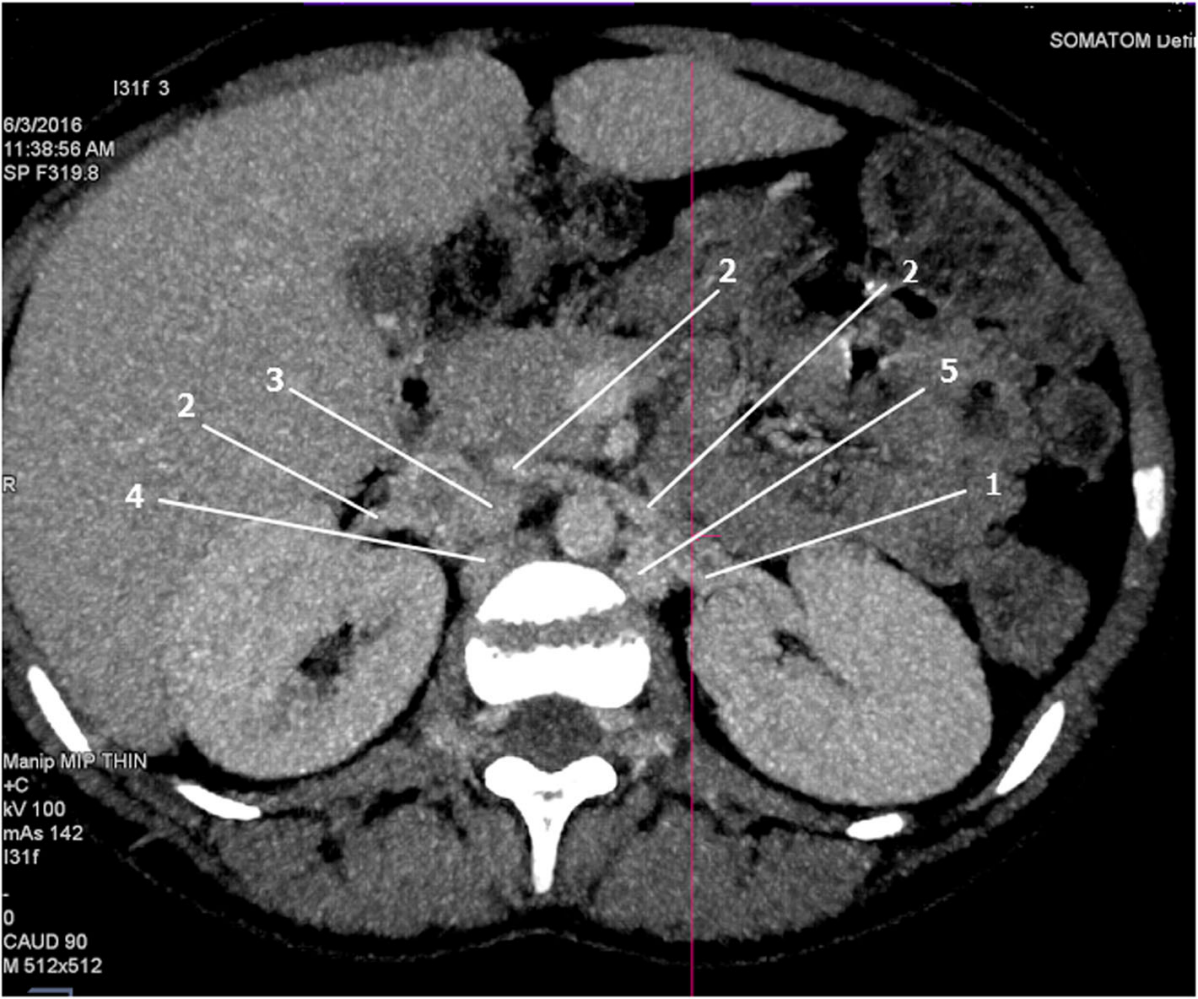
Computed tomography angiography (axial view) of the abdomen displaying posterior divisions of renal veins draining into the hemiazygos and azygos veins respectively. *1* posterior division of renal vein, *2* anterior division of renal vein, *3* inferior vena cava, *4* azygos vein, *5* hemiazygos vein

**Fig. 2:**
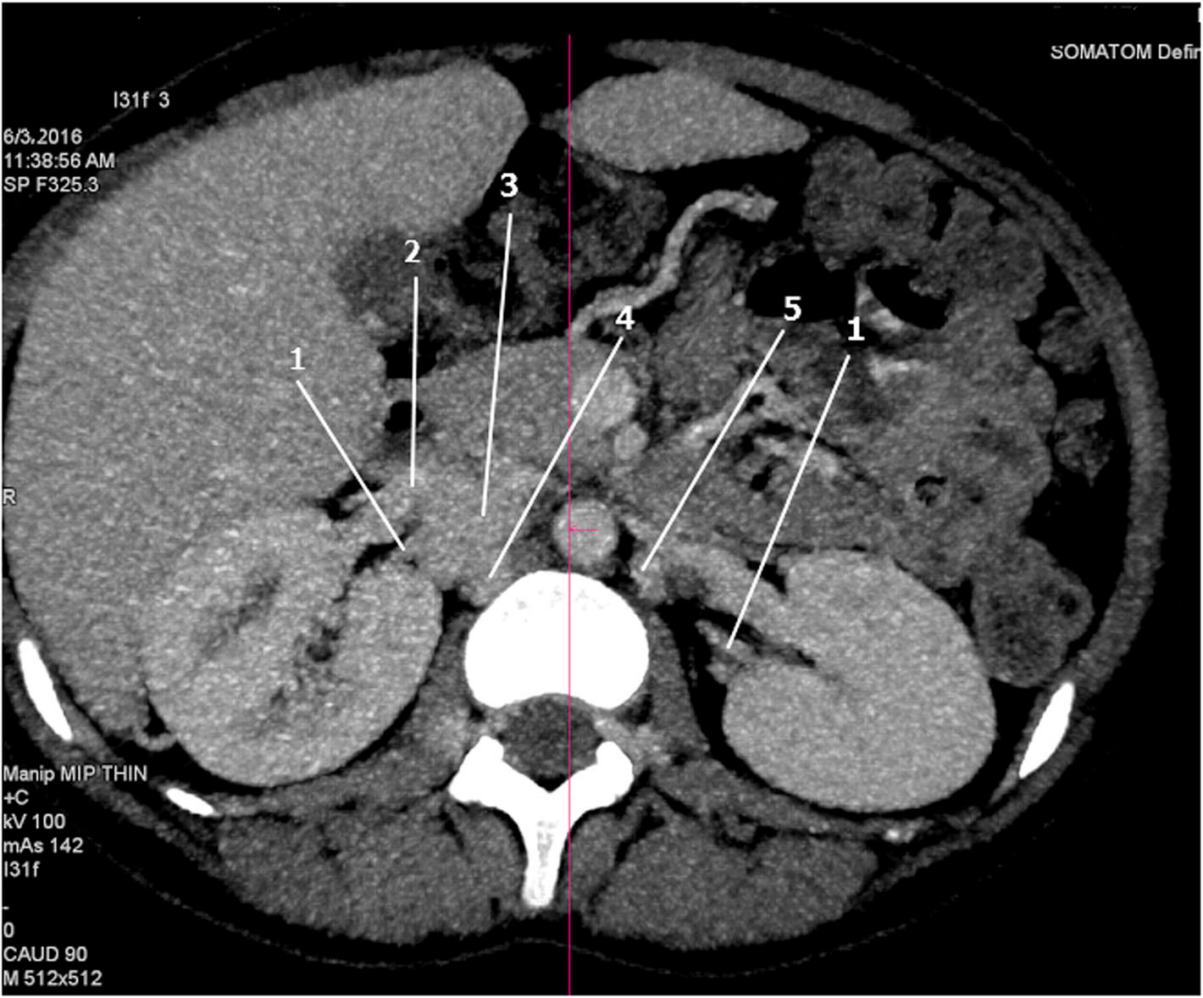
Computed tomography angiography (axial view) of the abdomen displaying posterior divisions of renal veins draining into the hemiazygos and azygos veins respectively. *1* posterior division of renal vein, *2* anterior division of renal vein, *3* inferior vena cava, *4* azygos vein, *5* hemiazygos vein

**Fig. 3:**
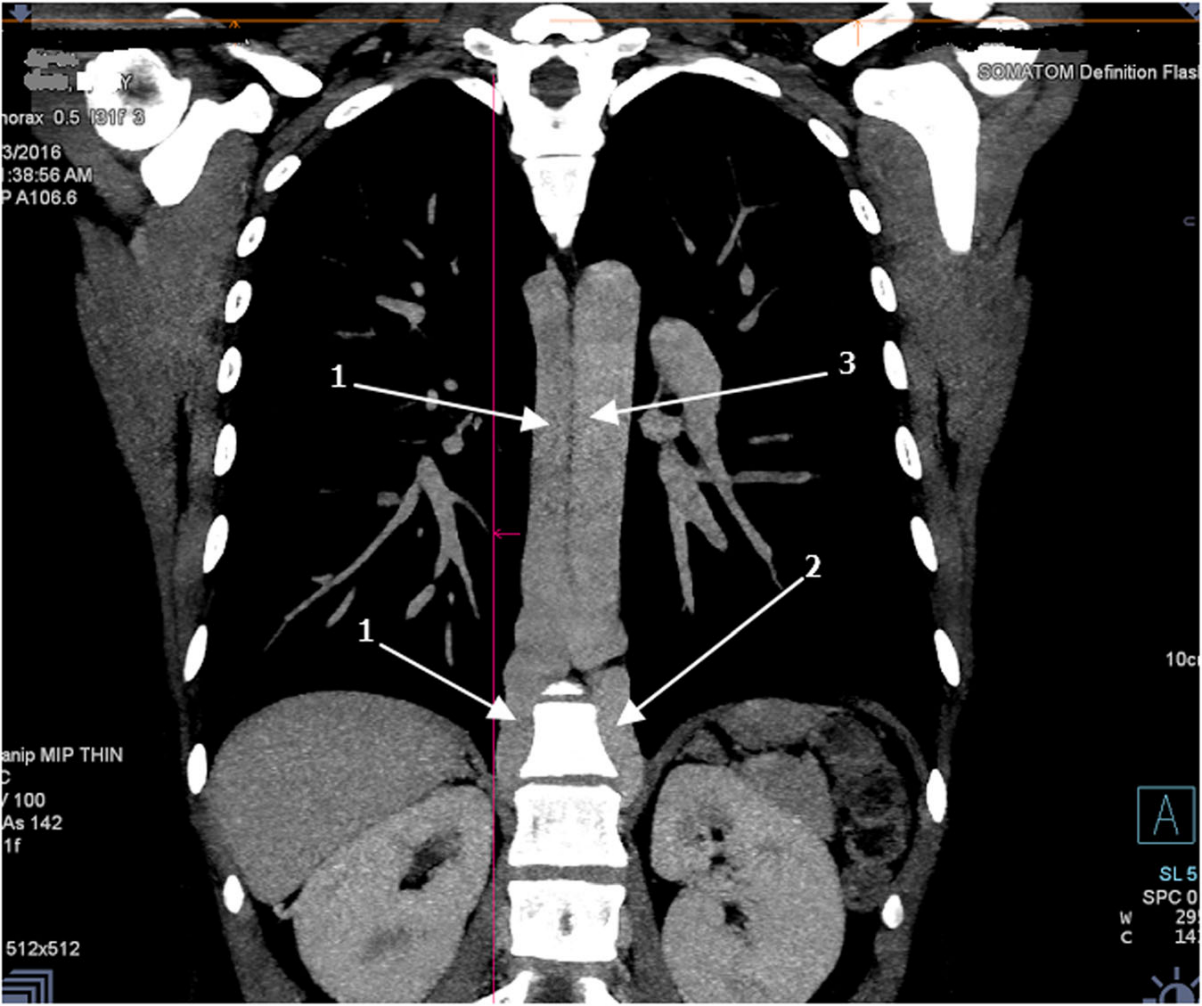
Computed tomography angiography (coronal view) of the thoracic region displaying tortuosity and dilatation of the azygos vein. *1* azygos vein, *2* hemiazygos vein, *3* aorta

A 20-year-old woman of African descent from Tanzania was referred to us from an upcountry hospital with a 12-year history of recurrent anemia accompanied by effort intolerance, palpitations, chest pain, and yellowish discoloration of sclera. She had been on long-term use of hematinics (folic acid and ferrous sulfate), had been hospitalized five times, and had been transfused three times in her lifetime. Her physical examination was unremarkable except for conjunctival pallor. Her vital signs during admission were blood pressure 98/54 mmHg, pulse rate 64 beats/minute, respiratory rate 18 breaths/minute, temperature 37.0 ^o^C, oxygen saturation 100% in room air, and she had a body mass index (BMI) of 20.5 kg/m^2^ [2]. She underwent several blood work-ups which revealed normal findings except for a normocytic normochromic anemia: hemoglobin (Hb) 8.88 g/dL, mean corpuscular volume (MCV) 91.2 fL, mean corpuscular hemoglobin (MCH) 30.5 pg/cell, and red cell distribution width (RDW) 21.6%. Her electrocardiogram (ECG) and echocardiography (ECHO) revealed normal findings. She had a positive sickling test and Hb electrophoresis revealed a sickle cell trait (AS). She also underwent computed tomography (CT) angiography and venography of her chest and abdomen to rule out the presence of arteriovenous malformations. Her aortography findings were normal but venography results revealed features of tortuously dilated azygos and hemiazygos veins each receiving blood from its respective posterior division of renal vein (Figs. 1, 2, and 3). Our patient and her family were counseled regarding the sickle cell trait and they were referred to a hematology out-patient clinic. Furthermore, the renal vein anomaly seen in CT was also communicated to our patient.

## Discussion

Variations in the renal vascular anatomy are rare and usually detected incidentally during a routine work-up. The advent of multi-detector computed tomography has increased the detection rate and improved the assessment experience of anatomic variations [6]. In general, persons with renal vein anomalies are asymptomatic but, rarely, they may present with a blunt pain, intermittent hematuria, and/or varicocele [6]. Preoperative knowledge of such variants is important in several angiographic procedures including adrenal or renal venography, renal vein sampling, and spermatic embolization [7]. Moreover, awareness of these anomalies is also crucial in retroperitoneal surgeries especially renal transplantation or when a distal portal decompression is anticipated [6, 7]. If unrecognized, these variations may lead to significant complications or death during abdominopelvic operations [4, 8].

In normal anatomy, the anterior and posterior divisions of renal veins receive blood from the anterior and posterior portions of the kidneys, respectively. The two divisions then unite to form a single renal vein that drains directly into the inferior vena cava (IVC) at a right angle on either side [4]. In the case presented, the anterior divisions drained normally into her IVC but the posterior divisions drained into the azygos and hemiazygos veins on the right and left side, respectively (Figs. 1 and 2). As a result, her azygos venous system was tortuous and dilated due to venous congestion (Fig. 3).

The clinical picture of vascular anomalies is extremely protean. These aberrant venous connections have the potential to cause venous congestion and compression and/or thrombosis with consequential hemodynamic compromises. In the case presented the anomaly was rather benign and had no contribution to the current symptomatology. However, despite it being an incidental finding, preoperative unawareness of the existence of such benign vascular anomalies can result in surgical trauma and its associated complications in the event that abdominopelvic surgery is indicated.

## Conclusions

Although venous anomalies are relatively infrequent and generally lack a clinical significance, a thorough understanding of embryologic development and its associated errors is of immense importance in equipping angiographers and surgeons to: (i) select appropriate interventional/operative techniques, (ii) anticipate risks and prevent intervention-related complications, and (iii) provide proper postoperative management.

## Abbreviations

BMI: Body Mass Index
CT: Computed tomography
ECG: Electrocardiogram
ECHO: Echocardiography
Hb: Hemoglobin
IVC: Inferior vena cava
MCH: Mean corpuscular hemoglobin
MCV: Mean corpuscular volume
RDW: Red cell distribution width
